# Real-World Persistency for Inflammatory Bowel Disease Biologics Using Patient Registry Data

**DOI:** 10.1093/crocol/otad051

**Published:** 2023-09-18

**Authors:** Tia Goss Sawhney, Angela Dobes, Sirimon O’Charoen

**Affiliations:** Teus Health, LLC, Newark, NJ, USA; New York University, School of Global Public Health, New York, NY, USA; Crohn’s & Colitis Foundation, New York, NY, USA; Crohn’s & Colitis Foundation, New York, NY, USA

**Keywords:** inflammatory bowel disease, Crohn’s disease, ulcerative colitis, biologic drugs, drug persistence, patient registry, drug discontinuation

## Abstract

**Background:**

Although it is a truism that drugs benefit patients only when taken, surprisingly little is known about real-world drug-use persistence and discontinuation, even for expensive biologic drugs.

**Methods:**

We used longitudinal self-reported drug-use data from the inflammatory bowel disease (IBD) Partners registry of people with IBD to construct Kaplan–Meier drug-use persistency graphs for biologic drug-use spans that started between 2017 and 2022.

**Results:**

We examined 2034 drug-use spans for 1594 survey participants. Most of the biologic drugs had a 75%+ persistency rate around the one-year mark and 60%+ persistency at the 3-year mark. The overall persistency and the differences in persistency between drugs were aligned with published literature.

**Conclusions:**

This analysis demonstrates the feasibility of collecting IBD-specific patient-reported drug persistency data via a voluntary patient registry. Patient-reported persistency provides real-world drug persistency data and the patient’s perspectives as to why they discontinued use of the drug—a combination of data and perspective that is not available from any other real-world medical record, claim, and pharmacy data source that are valuable to physician, patients, payers, healthcare policymakers, and health technology assessment organizations.

## Introduction

Although it is a truism that drugs benefit patients only when taken, surprisingly little is known about real-world drug-use continuation and discontinuation (persistence), even for expensive biologic drugs. This analysis will partially fill the persistency knowledge gap for the biologic drugs commonly used to treat people with Crohn’s disease and ulcerative colitis, the 2 major types of inflammatory bowel disease (IBD), and more generally demonstrate the power of patient-reported data for persistency studies. We use patient survey data from the IBD Partners registry, a patient-powered research network, and an online voluntary registry with more than 17,000 adult IBD patients from across the United States (US) and make recommendations as to how voluntary online patient registries may be best configured to provide reliable and generalizable drug-use persistence studies that can be used to support drug treatment and coverage decisions.

There is a need for real-world drug persistence studies. As physicians and payers make treatment and coverage decisions that impact those with chronic diseases, it is important to know what drugs are most likely to be taken long-term, may have the most side effects, and more. Because of the dearth of real-world studies, treatment, and coverage decisions often rely on clinical trials that describe the drug experience of small, curated populations of patients. Trial participants are actively encouraged to take the trial drug for the full duration of the trial (seldom more than 1 year) and are seldom followed thereafter. Furthermore, since each trial considers a unique drug, using trial-specific protocols, it is challenging to make drug-to-drug comparisons.

The Centers for Medicare and Medicaid Services (CMS), the agency responsible for Medicare and Medicaid coverage decisions, recently required real-world studies as a pre-requisite for the potential broad coverage of aducanumab (Aduhelm), a newly approved Alzheimer’s disease drug. Their April 2022 coverage decision for limited aducanumab coverage to Medicare beneficiaries was only for those participating in CMS-approved studies that collected data through routine clinical practice or registries. CMS noted that “registry data may be used to assess whether outcomes seen in carefully controlled clinical trials (e.g., FDA trials) are reproduced in the real-world and in a broader range of patients.”^1^

Health technology assessment organizations model the real-world cost-effectiveness of drugs over the lifespans of patients. Persistency is a key assumption within their models and their assessments, in turn, influence payer coverage decisions.

Real-world prescription drug data that can be used for persistent studies is available from several sources: Electronic health records, health insurance plans, prescription benefit managers, pharmacies, health insurance claims clearinghouses, and health data exchanges. These sources can be used to answer questions concerning what drugs were prescribed, filled, and/or administered. They typically cannot answer questions concerning whether these self-administered drugs were truly taken and why prescriptions were not filled, changed, or not renewed.

Patient-reported registry data have both advantages and disadvantages compared to these data sources. The major advantage is that patients know what drugs they took and why they stopped taking them. The disadvantage is that patient-reported data can be subject to recall biases, omissions, and other inaccuracies.^2,3^ (We are using the neutral terms of “persistence,” “continuation,” “discontinuation,” and “consistent use” instead of “adherence” or “compliance” as the latter terms imply that discontinuation and non-consistent use are universally the result of patients failing to follow medical advice.)

Patient-reported registry data is often directly collected from voluntary patients through surveys. Voluntary surveys, as a data collection tool, have limitations: The patients who elect to participate in surveys may not be representative of all real-world patients and patients in longitudinal surveys often discontinue their survey participation (are lost to follow-up). Representation and continuity limitations; however, are present to some degree for all data sources and collection tools. For example, health insurance claims data for commercially insured patients is not representative of Medicare and Medicaid patients and patients are often lost to follow-up when they change insurance.

## Data

We used deidentified IBD Partners registry data collected between 2011 and 2022.^4^ The registry is comprised of patient-reported data captured through a series of online surveys on topics such as diet, treatments, issues concerning patients’ disease management, quality of life, and drug use. The survey questions changed somewhat over time, but the biologic drug-use questions were consistent except for the addition of newly approved drugs. The surveys and data are available to researchers.^5^

The voluntary online registry was a joint project between the University of North Carolina School of Medicine and the Crohn’s & Colitis Foundation (Foundation). Participating patients voluntarily completed a survey twice a year. Participants initiated and discontinued survey participation at will and sometimes skipped one or several surveys. For their first survey, participants were asked to list their drugs and the start date for each drug. If the participant next responded to a survey, whether in 6 months or later, they were presented with their previously reported list of drugs and asked if they were still taking or had stopped the drugs. They were also asked for the start date, and if applicable the stop date, of any new drugs they had taken since their last survey. When a participant reported that they stopped taking a drug, they were asked why and responded by selecting a drop-down response. If their response was “other reason,” they were asked to elaborate.

The IBD Partners registry surveys asked identical medication usage questions for all Food and Drug Administration (FDA) approved IBD biologic drugs. These questions potentially allow for drug-to-drug comparisons for both Crohn’s disease and ulcerative colitis—an advantage of registry data relative to clinical trial data. Furthermore, US real-world persistency studies to date have typically examined 1 or 2 drugs for one IBD disease with each study using different data sources and methodologies. Khan, et al in their 2018 systematic review of IBD adherence and persistence observed that “Studies varied greatly in terms of their design, follow‐up times, study population, biologic agent studied and endpoint definitions, making across study comparisons challenging.”^6^

The most contemporary and comprehensive, US study of IBD biologic drug persistence is “Real-world Pattern of Biologic Use in Patients with Inflammatory Bowel Disease: Treatment Persistence, Switching, and Importance of Concurrent Immunosuppressive Therapy” by Chen, et al.^7^ This study examined biologic drug persistence for biologically naïve patients using Truven Health MarketScan®. We used this study to assess the credibility of the IBD Partners registry’s patient-reported data.

We also examined a recent study from the University of Messina that compared persistency rates between biologics; “Effectiveness and Safety Profiles of Biological Therapies in Inflammatory Bowel Disease: Real Life Data from an Active Pharmacovigilance Project” (2022) by Barbieri, et al.^8^ This Italian study, conducted between January of 2017 and December of 2021, examined the persistency of 5 biologics.

## Methodology

### Patient Consent and Institutional Review Board Approval

Participants gave their consent for the research use of their survey responses. The University of North Carolina provided the Institutional Review Board approval for the collection of the data and sharing of data with the Foundation. The Foundation has an Institutional Review Board exemption to use deidentified data to conduct this analysis.

### Time Period

We received all survey data since 2011 for deidentified participants who completed at least one survey between 2017 and 2022. The 2011 to 2016 data provided baseline data for participants and allowed us to determine 2017 + biologic drug naivety.

### Drugs

We examined persistence of 8 biologic drugs approved by the FDA for the treatment of Crohn’s disease and/or ulcerative colitis. These drugs and their FDA approval years are shown in Table 1. (Drugs were added to the survey upon FDA approval.)

**Table 1.** FDA-approved inflammatory bowel disease biologic drugs.

		FDA approval year^**^	
Biologic drug (brand name)	Drug class^9^	Crohn’s disease	Ulcerative colitis
Infliximab* (Remicade®)	Anti-TNF Agent	1998	2005
Adalimumab (Humira®)	Anti-TNF Agent	2007	2012
Certolizumab pegol (Cimzia®)	Anti-TNF Agent	2008	-
Natalizumab (Tysabri®)	Integrin Receptor Antagonist	2008	-
Golimumab (Simponi®)	Anti-TNF Agent	-	2013
Vedolizumab (Entyvio®)	Integrin Receptor Antagonist	2014	2014
Ustekinumab (Stelara®)	Interleukin-12 and -23 Antagonist	2016	2019
Risankizumab-rzaa (Skyrizi®)	Interleukin-23 Antagonist	2022	-

^*^This analysis includes later-approved infliximab biosimilars.

^**^Drugs approved for one disease are sometimes used off-label for the other.

### Drug-Use Span Creation

We created biologic drug-use spans for drug-use intervals that began between 2017 and 2022. A span is defined by a *start_date* and either a *stop_date* or a *last_use_survey_date.* We excluded spans where the *start_date* was more than 30 days prior to the date of the patient’s first survey. (The exclusion is to avoid continuity bias. The first survey only asked patients about the drugs that they were currently using and not about drugs that they had previously used and stopped. Therefore, if we reported those spans, we would only be reporting drug-use “successes”. Subsequent surveys, in contrast, asked about all drugs the patient had started since the previous survey, both the drugs they were still using and those that they had already stopped using.) We also excluded spans where the reported *stop_date* was prior to the *start_date*, spans where the patient was not a US resident, spans for patients whose IBD was neither Crohn’s disease nor ulcerative colitis (The survey allowed participation by patients with indeterminate colitis.), and spans for participants who reported impossibly overlapping drug usage periods. For example, the simultaneous use of 4 biologic drugs.

We assigned a *biologic_experience* status to each span based on the participant’s reported biologic history at the first survey and their biologic spans (or absence thereof) prior to the span’s *start_date*. For each *stop_date*, we captured the *stop_reason* and any write-in reason.

### Data Reporting

We developed summary statistics describing the spans and the demographics of the biologic drug-using participants. These demographics included their self-reported race, ethnicity, and how they were recruited into the registry.

### Kaplan–Meier Graphs

We constructed Kaplan–Meier (KM) graphs for drugs with at least 20 spans where survival was drug-use continuation, and the patient was lost to follow-up (censored) as of the *last_use_survey_date*. The KM graphs used a 30-day increment as a proxy for “one month.” KM graph lines were terminated when there were no longer at least 10 patients at risk of discontinuation. R version 4.1.2 with “Survival” and “Survminer” libraries were used to generate the KM graphs and to test for differences between drugs. We constructed separate graphs for Crohn’s disease and ulcerative colitis persistence for biologically naïve and biologically non-naïve patients. We used log-rank tests and compared survival curves to compare drug-to-drug KM persistency.^10^

### Stop Reason Analysis

Finally, we examined the distribution of *stop reasons* for consistency with the KM graphs. A priori we believed that drugs with notably higher discontinuation rates than other drugs should have more participants reporting non-effectiveness or complications as their *stop reason*.

Patients were asked if they discontinued due to “Side effect,” “Ineffectiveness,” “Completed treatment,” or “Other.” If the participant selected the “other” option, they were asked to write in a reason. We manually attributed some write-in reasons to “Completed treatment,” “Ineffectiveness,” and “Side effects.” We subcategorized the remaining write-ins. Our categorization of the write-in responses is detailed in the Supplement.

## Results

### Spans

There were 8150 participants who completed at least one survey between 2017 and 2022. They reported 2708 biologic drug start dates between 2017 and 2022. (Many people can manage IBD without biologics.) We applied the following exclusions to the biologic spans, in order:

1. *start_date* more than 30 days prior to first survey date (356).
2. stop_date before begin_date (57).
3. Non-US resident (143).
4. Reported IBD diagnosis of indeterminate colitis (91).
5. Impossibly overlapping spans (27).

These exclusion rules resulted in 2034 usable drug-use spans for 1594 unique participants.

### Participants

Of the 1594 unique participants, 1111 (70%) had Crohn’s disease and 483 (30%) had ulcerative colitis. Approximately 75% of the participants for both diseases were women. The average participant with Crohn’s disease was 47 years old at the *start_date* of their first span and had been 26.9 years old when first diagnosed with IBD. The average participant with ulcerative colitis was 50.6 years old at the *start_date* of their first span and had been 31.2 years old when first diagnosed.

Participants did not all respond to all the demographic questions. 861 (54.0%) of participants responded to race and ethnicity questions. Of those 18 (2.1%) identified as Black or African American and about 43 (2.8%) identified as Hispanic/Latino/Spanish origin. More than 75% of the IBD Partners participants had a college degree. These and additional participant demographics are shown in Table 2.

**Table 2.** Participant demographics.

Characteristic	Attribute	Total	Crohn’s disease	Ulcerative colitis
*All participants*				
Unique participants		1,594	1,111	483
Sex	Reported	99.9%	100.0%	99.8%
	Female	74.1%	73.6%	75.1%
	Male	25.9%	26.4%	24.9%
Age at first *start_date*	Average	48.1	47.0	50.6
Age when diagnosed	Reported	99.5%	99.4%	99.8%
	Average	28.3	27.0	31.2
Hispanic/Latino	Reported	97.1%	96.6%	98.3%
	Yes	2.8%	2.2%	4.2%
	No	97.2%	97.8%	95.8%
Race	Reported	54.0%	52.6%	57.4%
	White	93.6%	94.9%	91.0%
	Black/African American	2.1%	2.2%	1.8%
	Asian	1.3%	0.9%	2.2%
	Native Hawaiian/Other Pacific Islander	0.0%	0.0%	0.0%
	American Indian/Alaskan Native	0.1%	0.0%	0.4%
	More than one race	2.1%	1.7%	2.9%
	Other race	0.8%	0.3%	1.8%
Education	Reported	98.1%	97.8%	98.8%
	Less than 12th grade	0.9%	0.7%	1.3%
	12th grade	5.4%	6.1%	3.8%
	Some college	17.9%	19.0%	15.3%
	College degree	42.5%	40.6%	46.8%
	Graduate school	33.4%	33.6%	32.9%
Top States	Reported	99.9%	99.9%	99.8%
	New York	8.3%	7.7%	9.5%
	California	7.5%	6.8%	8.9%
	Massachusetts	5.5%	5.7%	5.0%
	Texas	5.3%	5.6%	4.6%
	Pennsylvania	5.0%	5.4%	4.1%
*Biologically Naïve Participants*				
Unique participants		770	466	304
Sex	Reported	99.9%	100.0%	99.7%
	Female	72.7%	71.9%	73.9%
	Male	27.3%	28.1%	26.1%
Age at first *start_date*	Average	47.0	47.3	46.6
Age when diagnosed	Reported	99.7%	99.6%	100.0%
	Average	30.1	29.1	31.6

Participants were primarily recruited via outreach from the Foundation. The top responses for the 982 participants who answered the “How did you hear about IBD Partners?” question were:

- 278 (28%) from the Foundation Website.
- 172 (18%) from Foundation emails.
- 146 (15%) from the Foundation help line.
- 66 (7%) from Foundation events.

Other methods of outreach included friends and family, media, social media, and internet postings.

The average participant had approximately 1.28 biologic drug-use spans. Multiple spans were mostly for multiple drugs, but sometimes participants stopped and restarted the same drug. Crohn’s disease participants had 1426 spans and participants with ulcerative colitis had 608 spans. The average reported time using a biologic drug (inclusive of loss to follow-up and censoring) was 22.6 months for Crohn’s disease participants and 18.5 months for ulcerative colitis participants. Biologic drug use spans are further detailed in Table 3.

**Table 3.** Biologic drug-use spans 2017 to 2022.

Disease	Drug name						
Crohn’s disease	All	Usteki- numab	Vedoli- zumab	Adali- mumab	Inflix- imab	Certoli- zumab	All Other
Unique participants	1,111	572	300	254	192	67	23
Biologic drug spans	1,426	581	303	258	193	67	24
Percent biosimilar spans	3.1%	0.0%	0.0%	0.0%	22.8%	0.0%	0.0%
Spans per participant	1.28	1.02	1.01	1.02	1.01	1.00	1.04
Biologic naïve spans	466	129	94	135	89	16	3
Avg *begin_date*	Apr-2019	Jun-2019	May-2019	Jan-2019	Feb-2019	Feb-2019	-
Avg months from begin date to *last_date*	22.6	23.7	22.7	23.3	22.2	13.6	-
Percent stopped	29.7%	22.2%	32.7%	33.7%	33.2%	53.7%	-
Percent lost to follow-up	34.5%	37.9%	31.7%	33.3%	34.7%	28.4%	-
Percent lost to study termination	35.8%	39.9%	35.6%	33.0%	32.1%	17.9%	-
Ulcerative colitis	All	Usteki- numab	Vedoli- zumab	Adali- mumab	Inflix- imab	Goli- mumab	All other
Unique participants	483	124	225	123	104	21	8
Biologic drug spans	608	126	225	123	105	21	8
Percent biosimilar spans	4.3%	0.0%	0.0%	0.8%	23.8%	0.0%	0.0%
Spans per participant	1.26	1.02	1.00	1.00	1.01	1.00	1.00
Biologic naïve spans	304	26	112	84	73	6	3
Avg *begin_date*	Aug-2019	Jul-2020	Jun-2019	Mar-2019	May-2019	Jan-2019	-
Avg months from begin date to *last_date*	18.5	17.3	20.8	15.5	20.5	13.0	-
Percent stopped	32.7%	24.6%	24.0%	49.6%	31.4%	76.2%	-
Percent lost to follow-up	34.4%	27.8%	42.2%	31.7%	34.3%	14.3%	-
Percent lost to study termination	32.9%	47.6%	33.8%	18.7%	34.3%	9.5%	-

[1] Golimumab (15), natalizumab (3), and risankizumab-rzaa (6).

[2] Certolizumab (7), risankizumab-rzaa (1).

nr = not reported.

Lost to study termination =* last_use_survey_date* was between July and December 2022.

Last_date = stop_date or last_use_survey_date.

### Kaplan–Meier Graphs

**Figure 1.**
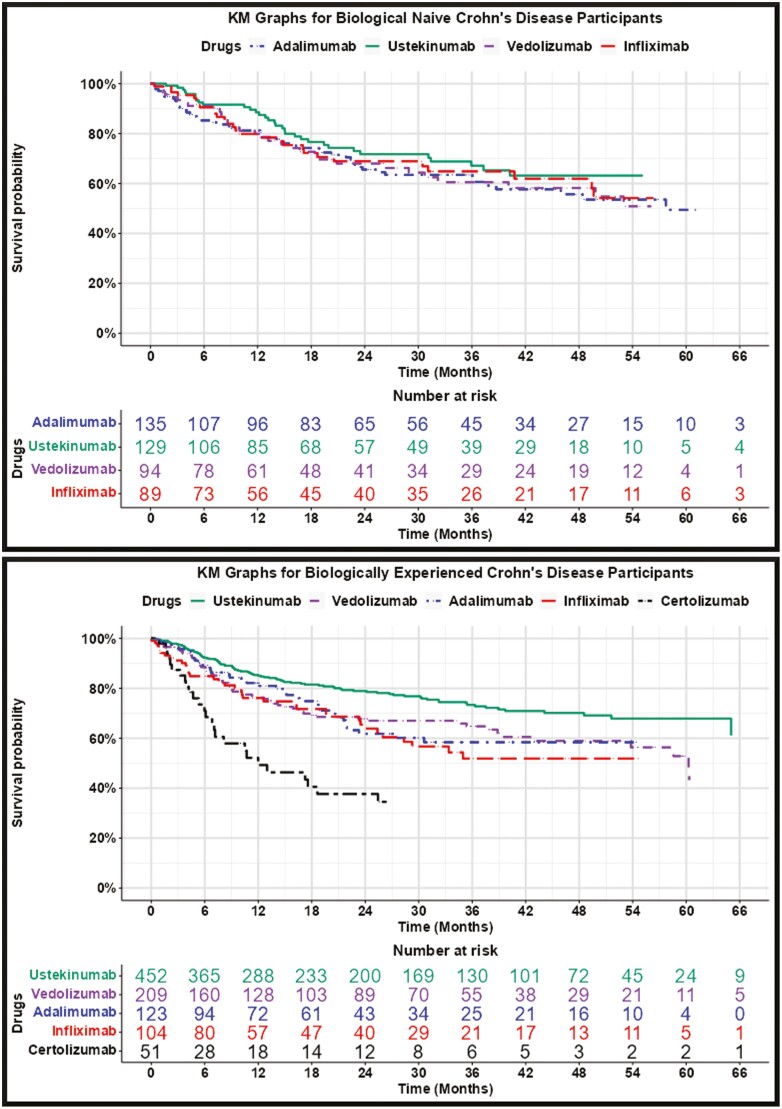
Crohn’s Disease Kaplan–Meier Graphs. *Note: Kaplan–Meier graphs are shown only for drugs with 20 + initial spans and are discontinued when there are less than 10 spans.*

**Figure 2.**
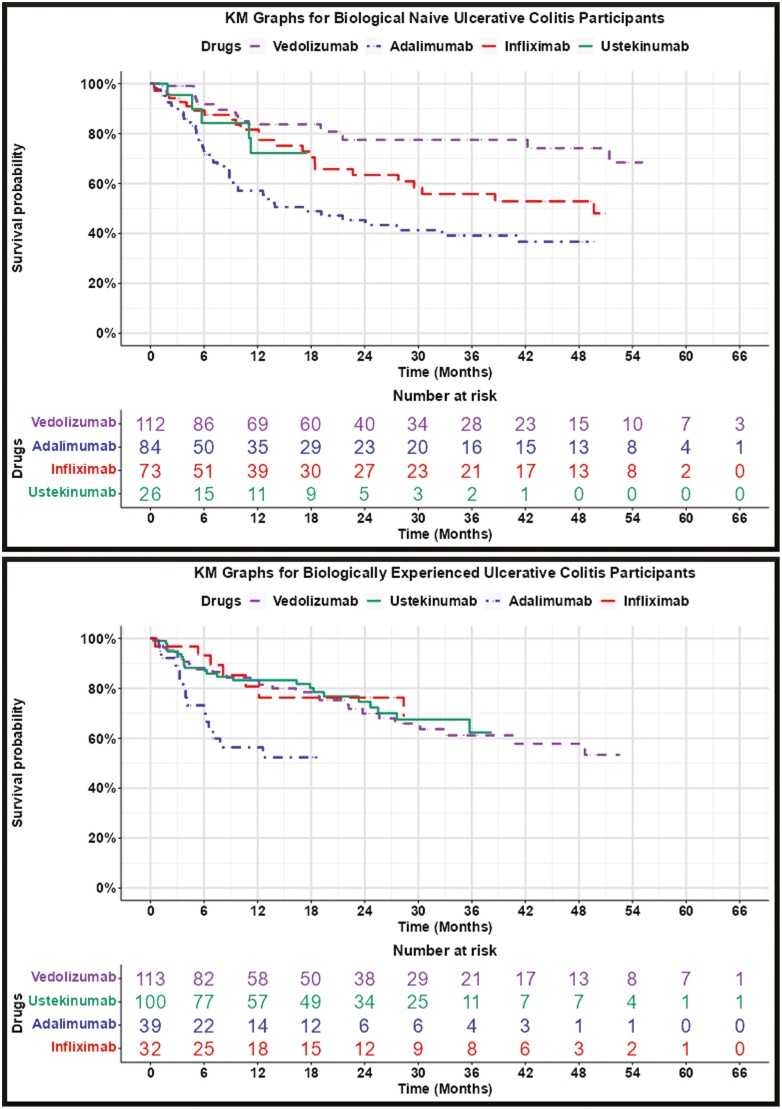
Ulcerative Colitis Kaplan–Meier Graphs. *Note: Kaplan–Meier graphs are shown only for drugs with 20 + initial spans and are discontinued when there are less than 10 spans.*

The KM graphs provided in Figures 1 and 2, and summarized in Tables 4 and 5, show similar persistence across IBD drugs, with some exceptions:

**Table 4.** Kaplan–Meier Summaries.

Crohn’s disease					Ulcerative colitis				
Drug	Number of spans	Persistency at end of month			Drug	Number of spans	Persistency at end of month		
		12	24	36			12	24	36
Biologically naïve participants					Spans for biologically naïve participants				
Adalimumab	135	81.2%	65.6%	63.5%	Vedolizumab	112	83.7%	77.5%	77.5%
Ustekinumab	129	88.5%	71.8%	67.1%	Adalimumab	84	57.1%	45.3%	39.1%
Vedolizumab	94	79.8%	68.0%	60.5%	Inflximab	73	81.6%	63.4%	55.8%
Infliximab	89	79.9%	68.9%	64.9%	Ustekinumab	26	72.2%	*not reported*	
Certolizumab	<20	*not reported*			Golimumab	<20	*not reported*		
Biologically experienced participants					Spans for biologically experienced participants				
Ustekinumab	452	85.0%	78.9%	73.4%	Vedolizumab	113	82.9%	69.9%	61.2%
Vedolizumab	209	76.3%	67.8%	64.8%	Ustekinumab	100	83.2%	74.6%	62.3%
Adalimumab	123	82.1%	61.8%	58.4%	Adalimumab	39	56.4%	*not reported*	
Infliximab	104	76.1%	63.9%	51.9%	Infliximab	32	80.8%	76.3%	*not reported*
Certolizumab	51	49.2%	37.7%	*not reported*	Golimumab	<20	*not reported*		

Note: Kaplan–Meier graph data are reported only for drugs with 20 + initial spans and are discontinued when there are less than 10 spans.

**Table 5.** Kaplan–Meier Statistical Testing.

Crohn’s disease *P*-values						Ulcerative colitis *P*-values					
Biologically naïve participants	Adalimumab	Ustekinumab	Vedolizumab	Infliximab	Certolizumab	Biologically naïve participants	Vedolizumab	Adalimumab	Infliximab	Ustekinumab	Golimumab
Adalimumab	na	0.67	0.91	0.91	not reported	Vedolizumab	na	0.00	0.02	0.49	*not reported*
Ustekinumab	0.67	na	0.67	0.81		Adalimumab	0.00	na	0.09	0.09	
Vedolizumab	0.91	0.67	na	0.91		Infliximab	0.02	0.09	na	0.62	
Infliximab	0.91	0.81	0.91	na		Ustekinumab	0.49	0.09	0.62	na	
Certolizumab	*not reported*				na	Golimumab	*not reported*				na
Biologically experienced participants	Ustekinumab	Vedolizumab	Adalimumab	Infliximab	Certolizumab	Biologically experienced participants	Vedolizumab	Ustekinumab	Adalimumab	Infliximab	Golimumab
Ustekinumab	na	0.01	0.03	0.00	0.00	Vedolizumab	na	0.86	0.00	0.67	*not reported*
Vedolizumab	0.01	na	0.90	0.54	0.00	Ustekinumab	0.86	na	0.00	0.67	
Adalimumab	0.03	0.90	na	0.54	0.00	Adalimumab	0.00	0.00	na	0.01	
Infliximab	0.00	0.54	0.54	na	0.00	Infliximab	0.67	0.67	0.01	na	
Certolizumab	0.00	0.00	0.00	0.00	na	Golimumab	*not reported*				na

KM graphs statistical tests are shown for drugs with 20 + initial spans. The tests used all data points including months when there were less than 10 spans.

- Ustekinumab for biologically experienced Crohn’s patients had statistically significant (*P*-value < .05) higher persistence than all other drugs.
- Adalimumab for biologically naïve and biologically experienced ulcerative colitis patients had statistically significantly lower persistence compared with other drugs.
- Certolizumab pegol for biologically experienced Crohn’s disease patients had low use and statistically significantly lower persistence than all other drugs. (The use of certolizumab pegol for biologically naïve Crohn’s disease was too low to report.)
- Golimumab for ulcerative colitis was not used often enough to be able to report experience for biologically naïve and biologically experienced patients.

Ustekinumab’s statistically higher persistence for Crohn’s disease was muted and not statistically significant for biologically naïve spans. Adalimumab’s statistically lower persistence for ulcerative colitis was also apparent for biologically naïve spans and was statistically significant in comparison to vedolizumab.

We anticipated a positive relationship between drug efficacy, the number of people starting a drug, and persistence. Table 4 shows a positive relationship between the number of drug-use spans and drug persistence: The more frequently used drugs generally have higher persistence. Table 4 also shows relative volumes of use and comparative persistency consistent with efficacy studies and expert opinion:

- Real-world clinical studies have demonstrated ustekinumab’s superiority over anti-TNF drugs for anti-TNF experienced (non-biologic naïve) Crohn’s disease patients.^11^
- A 2015 network meta-analysis of infliximab, adalimumab, and golimumab for ulcerative colitis found that “infliximab was statistically superior to adalimumab after induction.”^12^
- A 2015 expert opinion piece testing the efficacy of certolizumab pegol found a “lack of clear benefit in key outcomes of inducing remission and mucosal healing.”^13^
- A 2022 systemic review and meta-analysis testing the efficacy and safety of biologics and small molecule drugs that included golimumab found that “all interventions but fligotinib 100mg, golimumab, and erlizumab were significantly superior to placebo.”^14^

### Reason for Discontinuation

Ineffectiveness was the most common reason participants discontinued a biologic for Crohn’s disease and ulcerative colitis. The ineffectiveness could be due to the drug not inducing an initial response, loss of response after multiple doses, or a participant developing antibodies to a biologic (Immunogenicity).^7^ The second most common reason among participants for discontinuing a biologic was unwanted side effects of the drug. Antibody development was a common “Other” write-in reason for discontinuation. There are undoubtedly other instances of antibody development buried in “Side effect” and “Ineffectiveness.”

The discontinuation reasons were relatively similar across drugs, except for Infliximab for Crohn’s disease, where side effects were responsible for 44% of the discontinuations—much higher than for the other drugs. Furthermore, another 8% of the terminations were for “Other: antibody development,” also much higher than for other drugs.

Less than 5% of discontinuations were attributed to cost or insurance. See Table 6 for discontinuation reasons.

**Table 6.** Discontinuation reasons.

Disease	Drug name						
Crohn’s disease	All	Usteki- numab	Vedoli- zumab	Adali- mumab	Inflix- imab	Certoli- zumab	All other
Side effect	21.5%	13.2%	19.2%	24.1%	43.8%	13.9%	11.1%
Ineffectiveness	60.1%	65.1%	67.7%	58.6%	34.4%	69.4%	66.7%
Completed treatment	2.6%	4.7%	2.0%	0.0%	3.1%	0.0%	11.1%
Cost/insurance issues	3.8%	6.2%	0.0%	6.9%	1.6%	2.8%	0.0%
Other	12.0%	10.9%	11.1%	10.3%	17.2%	13.9%	11.1%
Antibody development	1.7%	0.0%	0.0%	0.0%	7.8%	2.8%	11.1%
Comorbid autoimmune disorder/cancer tx	1.7%	2.3%	3.0%	0.0%	0.0%	2.8%	0.0%
Immunosuppression	1.7%	0.0%	0.0%	5.7%	3.1%	0.0%	0.0%
Other specified	0.7%	1.6%	1.0%	0.0%	0.0%	0.0%	0.0%
Rx change—reason unspecified	0.9%	0.8%	1.0%	0.0%	1.6%	2.8%	0.0%
Surgery/pregnancy	2.6%	3.1%	3.0%	2.3%	1.6%	2.8%	0.0%
Unspecified	2.8%	3.1%	3.0%	2.3%	3.1%	2.8%	0.0%
Total	424	129	99	87	64	36	9

Ulcerative colitis	All	Usteki- numab	Vedoli- zumab	Adali- mumab	Inflix- imab	Goli- mumab	All other
Side effect	13.6%	9.7%	14.8%	13.1%	18.2%	12.5%	0.0%
Ineffectiveness	70.4%	77.4%	66.7%	68.9%	75.8%	75.0%	25.0%
Completed treatment	2.5%	3.2%	3.7%	3.3%	0.0%	0.0%	0.0%
Cost/insurance issues	2.5%	0.0%	3.7%	1.6%	3.0%	0.0%	25.0%
Other	11.1%	9.7%	11.1%	13.1%	3.0%	12.5%	50.0%
Antibody development	0.5%	0.0%	0.0%	1.6%	0.0%	0.0%	0.0%
Comorbid autoimmune disorder/cancer tx	2.0%	3.2%	1.9%	0.0%	0.0%	6.3%	25.0%
Immunosuppression	0.0%	0.0%	0.0%	0.0%	0.0%	0.0%	0.0%
Other specified	2.0%	3.2%	1.9%	0.0%	0.0%	6.3%	25.0%
Rx change—reason unspecified	2.0%	0.0%	3.7%	3.3%	0.0%	0.0%	0.0%
Surgery/pregnancy	3.0%	0.0%	3.7%	4.9%	3.0%	0.0%	0.0%
Unspecified	1.5%	3.2%	0.0%	3.3%	0.0%	0.0%	0.0%
Total	199	31	54	61	33	16	4

Antibody development = participant clearly cited antibody development.

Comorbid autoimmune disorder/cancer tx = participant said another drug was a better fit with their comorbid autoimmune disease.

or cancer treatment.

Immunosuppression = participant had a condition associated with immunosuppression or was concerned about immunsuppression.

Rx change—reason unspecified = participant verified a move to another drug, but did not provide a reason.

Surgery/pregnancy = participant pregnant or planning pregnancy or surgery.

## Discussion

### Comparison to Other Papers

Chen et al studied biologically naïve patients and found that persistency for biologics for both Crohn’s disease and ulcerative colitis was on average below 50% at the end of the first year. Our analysis shows that while the lower-persistency biologics dropped to near or below 50% persistence at 1-year, most of the biologics had a 75%+ persistency rate at 1-year and 60%+ persistency at 3-years.

Like us, Chen et al found particularly lower persistency for certolizumab pegol, a less commonly used drug. (Ustekinumab was not approved at the time of the Chen study.)

The difference in persistency between Chen et al and our analysis could be the result of (1) the populations studied, (2) the time periods studied, (3) the definition of persistency, and/or (4) persistency bias in patient-reported data.

- *Populations*. There are substantial differences in the populations. Chen et al examined from 2008 to 2015 health insurance claims data for people with employer-sponsored insurance. They classified an insured as biologically naïve if the insured had not had a biologic drug at any time in the prior 12 months. Their population was 20% children and nearly 50% male. Our biologically naïve population is, in contrast, spans insurance types, is truly biologically naïve, all adult, and only 27% male.
- *Time periods*. The drug-use spans Chen et al created were for 2009–2015. Our spans were for 2017–2022. Over these years various new treatments entered the market, ongoing vedolizumab treatments moved from intravenous infusion to injections,^15^ mail order drug delivery and home infusions became more common,^16^ and pharmacies and physicians started delivering text and other electronic reminders. These changes were intended to improve drug persistency.
- *Definition of persistency*. Chen et al identified patients who stopped a drug as those with “a drug-[refill]-free period greater than the days-of-supply of previous administration.”^7^ This definition did not allow for delayed or missed doses. In additon, Chen’s definition of naivety required 12 prior months of no IBD diagnoses or IBD-related biologic drugs. We, in contrast, required no prior use of biologic drugs and relied upon survey participants to tell us when they stopped taking a drug. Our patient-reported drug-use periods therefore include time periods with delayed or missed doses. For example, a participant who between surveys missed a month or 2 of drugs while getting insurance company approval will likely have answered “yes” when asked “are you continuing to take the drug?” They never stopped; their refill was delayed.
- *Persistency bias in patient-reported data*. There is likely a correlation between IBD patients who are persistent in taking their drugs and IBD patients who are persistent in participating in the IBD Partners registry.

Results in this analysis, however, are similar to the Barbieri et al study on the 2017–2021 persistency of biologics among Italians. The Barbieri et al study compared 5 biologics: Infliximab, adalimumab, golimumab, vedolizumab, and ustekinumab. At the 1-year mark, the persistency rate for these 5 biologics was generally 75%+ at 1-year and 55%+ at 3-years.

Overall, we feel that our results are in general alignment with the Chen and Barbieri studies.

### Cost/Insurance

Biologics are expensive drugs, generally unaffordable out-of-pocket, and it is often a cumbersome process to get insurance plan or PBM approval for their use—an approval that must be periodically renewed. Yet less than 5% of discontinuation reasons were attributed to insurance or cost. This may be because the insurance/PBM approval process more often denies or delays drug initiation or delays ongoing authorization rather than forcing drug discontinuation.^17^ Furthermore, there are various patient assistance programs that fully cover or subsidize out-of-pocket costs.^18^

### Limitations

We believe that IBD Partners data fairly represents the drug persistency of the people participating in the online registry. The drug persistency of these participants, however, is not necessarily generalizable to all IBD patients. Compared to all IBD patients, the participants underrepresented men, Hispanics, and Blacks, and young people relative to estimates of the total US IBD population. A 2018 Centers for Disease Control morbidity and mortality weekly report estimated that between 2015 and 2016, a little over 3.1 million Americans self-reported that they had ever received a diagnosis of IBD. Of those that were diagnosed, approximately 61% were women, 76% were White, 5.6% were Black, 13.7% were Hispanic, and 5% were Non-Hispanic Other.^19^ The participants likely underrepresented the non-college-educated IBD population as more than 75% of the IBD participants had a college degree and in 2021 only 50% of US adults had completed college or graduate school.^20^ IBD is prevalent among children, who are not permitted to participate in the adult-only IBD Partners registry.^21^

As a result of their education, the participants are also likely wealthier and more engaged and activated in the management of their healthcare than the average IBD patient. Participation in the registry is, itself, a marker of patient engagement and activation.^22^

### Recommendations

It is important for research generated from online direct-to-patient registries to be representative of the entire patient population. Strategies to increase diversity include the identification of strategic recruitment partnerships to build trusted relationships with organizations that serve diverse and underrepresented communities to encourage less-represented patients to join the registry. In addition, when possible, registries should seek to partner with providers and insurers who serve diverse populations of people with IBD, including people with Medicaid coverage—all of whom are low-income.

Although drug-use data were systematically collected, drug-use was never a central focus of the IBD Partners data collection and studies. Opportunities to enhance IBD Partners registry design to better support drug-use studies include:

- *Real-time data edits.* There should be data edits to prevent entering a stop date that is before the (previously reported) start date of a drug and reporting the simultaneous use of an impossible number of drugs.
- *Insurance information*. It would be helpful to have insurance information for the participants.
- *Diagnosis verification*. To increase the reliability of the data, incorporation of a process to confirm self-reported diagnosis information would be valuable.
- *Dosage data*. IBD patients often need to take increasing doses of biologics over time or if they have particularly severe disease. It would be useful to collect dosage data.
- *Real-time, event-based data collection*. IBD Partners collected data on a 6-month cadence. To better understand if there were periods of temporary treatment stoppage due to insurance coverage delays it would be beneficial to enable a mechanism for patients to report on events in real time such as a treatment delay due to dose change. This could also help with recall bias concerns.

### Future Endeavors

In future analyses, it would be useful to examine how persistency rates are affected when patients are on a combination of immunomodulators, such as azathioprine, mercaptopurine, or methotrexate, and biologics. It would also be valuable to explore inconsistent drug use (delayed or missed or delayed doses) and the relationship between consistency, persistency, and patient symptoms and quality of life.

## Conclusions

We used real-world, patient-reported registry data to examine differences in IBD biologic drug persistency and overall persistency. The differences we found aligned with reported clinical and real-world efficacy from published literature. And the overall persistency rates aligned with other persistency studies.

We believe that drug-use persistence is best understood from the perspective of patients. This analysis demonstrates the feasibility of collecting IBD-specific patient-reported persistence data via a voluntary patient registry. Patient-reported persistency provides real-world drug persistency data and the patients’ perspectives as to why they discontinued use of the drug—a combination of data and perspective that is not available from any other real-world medical record, claim, and pharmacy data source that are valuable to physician, patients, payers, healthcare policymakers, and health technology assessment organizations.

## Supplementary Material

otad051_suppl_Supplementary_Tables_1

## Data Availability

The IBD Partners data are available upon approved application to the Crohn’s & Colitis Foundation’s IBD Plexus program (https://www.crohnscolitisfoundation.org/ibd-plexus).
