# Supplementary material for: Real-World Persistency for Inflammatory Bowel Disease Biologics Using Patient Registry Data
Source: Crohns Colitis 360. 2023 Sep 18;5(4):otad051. doi: 10.1093/crocol/otad051 (PMC10629214; doi:10.1093/crocol/otad051)
Supplement: otad051_suppl_Supplementary_Tables_1 [file otad051_suppl_supplementary_tables_1.docx]

**Real-World Persistency for** **IBD Biologics Using Patient Registry Data**

**Supplementary Data**

**Supplementary Table 1: Categorization of Patient Write-in Responses for “Other” Stop Reasons**

| **Patient Write-in Response for “Other” Stop Reason** | **Attributed Category** | **Attributed Subcategory** |
| --- | --- | --- |
| Felt fine | Completed treatment |  |
| Not indicated after resection | Completed treatment |  |
| Didn't want to take anymore | Completed treatment |  |
| Thought I was in permanent remission. | Completed treatment |  |
| Looking for better control of PsA | Ineffectiveness |  |
| In preparation for a drug trial; and due to ineffe | Ineffectiveness |  |
| Made Crohns Flare for 2 weeks | Ineffectiveness |  |
| Drug no longer effective | Ineffectiveness |  |
| Allergic reaction | Side effect |  |
| Arthritis pain | Side effect |  |
| Drug induced Lupus | Side effect |  |
| Allergic reaction | Side effect |  |
| Reaction | Side effect |  |
| Reaction with combo therapy | Side effect |  |
| Allergic reaction | Side effect |  |
| Adverse reaction of ITP | Side effect |  |
| Angioedema from it | Side effect |  |
| Nephrotic syndrome | Side effect |  |
| Trouble with veins | Side effect |  |
| Antibodies to the medication | Other | Antibody development |
| Antibodies | Other | Antibody development |
| Antibodies | Other | Antibody development |
| Body built up antibodies to Remicade | Other | Antibody development |
| Antibodies | Other | Antibody development |
| Antibodies | Other | Antibody development |
| Antibody development | Other | Antibody development |
| Antibodies | Other | Antibody development |
| Not effective for arthritis (worked for Crohn's) | Other | Comorbid autoimmune disorder/cancer tx |
| Ineffective for RA | Other | Comorbid autoimmune disorder/cancer tx |
| Using methotrexate for lymphoma | Other | Comorbid autoimmune disorder/cancer tx |
| Ineffective for ankylosis game spondylitis | Other | Comorbid autoimmune disorder/cancer tx |
| Needed Humira for another autoimmune condition | Other | Comorbid autoimmune disorder/cancer tx |
| Taking prednisone with my lymphoma therapy | Other | Comorbid autoimmune disorder/cancer tx |
| Needed joint coverage in addition to gut coverage | Other | Comorbid autoimmune disorder/cancer tx |
| Uveitis still occurring | Other | Comorbid autoimmune disorder/cancer tx |
| Needed something to help with my uveitis | Other | Comorbid autoimmune disorder/cancer tx |
| Pyoderma Gangrenosum not responsive | Other | Comorbid autoimmune disorder/cancer tx |
| Didn't address arthritis | Other | Comorbid autoimmune disorder/cancer tx |
| Switched to Pentasa due to Covert 19 | Other | Immunosuppression |
| Covid 19 risk due to immunity suppression | Other | Immunosuppression |
| UTI | Other | Immunosuppression |
| Positive Tb test | Other | Immunosuppression |
| Increased risk of more recurrent cancers. | Other | Immunosuppression |
| Developed peristomal pyoderma after surgery and it | Other | Immunosuppression |
| Was on Enbrel - stopped due to multiple infections | Other | Immunosuppression |
| Change medication | Other | Rx change - reason unspecified |
| Switched to Humira | Other | Rx change - reason unspecified |
| Not sure, may be raising eosinophils | Other | Rx change - reason unspecified |
| Changed meds | Other | Rx change - reason unspecified |
| Switch to Infusion Remicade | Other | Rx change - reason unspecified |
| Did not use it | Other | Rx change - reason unspecified |
| Wanted to try holistic medicine | Other | Rx change - reason unspecified |
| Wanted to try Stelara | Other | Rx change - reason unspecified |
| Surgery | Other | Surgery/Pregnancy |
| Planning for surgery. Surgeon asked that I stop Hu | Other | Surgery/Pregnancy |
| Was advised to stop before surgery | Other | Surgery/Pregnancy |
| Had to stop prior to,surgery | Other | Surgery/Pregnancy |
| Surgery Pause | Other | Surgery/Pregnancy |
| paused for surgery & covid vaccine | Other | Surgery/Pregnancy |
| Surgical prep | Other | Surgery/Pregnancy |
| Surgery prep | Other | Surgery/Pregnancy |
| Stopped due to surgeries | Other | Surgery/Pregnancy |
| Pending surgery | Other | Surgery/Pregnancy |
| Intestnal surgery | Other | Surgery/Pregnancy |
| Total colectomy | Other | Surgery/Pregnancy |
| Prep for surgery for colon removal | Other | Surgery/Pregnancy |
| Colon removed | Other | Surgery/Pregnancy |
| Pregnancy | Other | Surgery/Pregnancy |
| Surgery | Other | Surgery/Pregnancy |
| Got pregnant | Other | Surgery/Pregnancy |
| On hold | Other | Other specified |
| Covid pneumonia intubated. | Other | Other specified |
| Inconvenience | Other | Other specified |
| Neurological issues currently under evaluation | Other | Other specified |
| Dr. and I are trying to control with diet | Other | Other specified |
| Not primary reason for healing | Other | Other specified |
| Discontinued after Cytoxan during stem cell harves | Other | Other specified |
